# Effect of Microstructure on the Corrosion Resistance of TIG Welded 1579 Alloy

**DOI:** 10.3390/ma12162615

**Published:** 2019-08-16

**Authors:** Andrey S. Gnedenkov, Sergey L. Sinebryukhov, Dmitry V. Mashtalyar, Igor M. Imshinetskiy, Igor E. Vyaliy, Sergey V. Gnedenkov

**Affiliations:** 1 Institute of Chemistry of FEB RAS, 159 Pr. 100-letiya Vladivostoka, Vladivostok 690022, Russia; 2 School of Engineering, Far Eastern Federal University, 8 Sukhanova St., Vladivostok 690950, Russia

**Keywords:** aluminum alloy, welding, localized corrosion, intermetallic compounds, protective coating

## Abstract

The paper studies microstructure, chemical composition and corrosion activity of the tungsten inert gas welded joint of the Al-Mg-Sc alloy. An intensive corrosion attack of the heat affected zone (HAZ) was found due to precipitation of secondary phases at recrystallized grain boundaries. The ccorrosion process initiated along the boundary of α-Al grains, where a high concentration of anodic (Mg_2_Si and Mg_2_Al_3_) and cathodic phases ((MnFe)Al_6_) was observed. Increased temperatures during welding led to coalescence of the anodic phases in HAZ. Additionally, HAZ was found to be enriched with hard intermetallic compounds (Mg_2_Si and (MnFe)Al_6_). This area had a higher microhardness (930 MPa) compared to base metal (BM, 895 MPa) and fusion zone (FZ, 810 MPa). The volume fraction of secondary phases was 26% in BM, 28% in FZ and 38% in HAZ. The average grain size increased in the following order: (9 ± 3) µm (BM) < (16 ± 3) µm (HAZ) < (21 ± 5) µm (FZ). A plasma electrolytic oxidation (PEO) coating of aluminum-based material was applied to protect the weld from oxidation. The PEO-coating provided a high corrosion protection in the aggressive Cl^−^-containing environment.

## 1. Introduction

Currently, aluminum alloys remain one of the main structural materials for products manufactured by enterprises of various industries e.g.: aerospace, automobile, and marine. However, the presence of intermetallic phases with different corrosion potential in the aluminum alloys activates the localized corrosion and accelerates the material degradation [1,2,3,4]. Therefore, there are works dealt with study the corrosion activity of aluminum-based intermetallic phases [5,6,7,8].

Nowadays in the aerospace industry to increase the fuel efficiency of the product, which has a specific application, the weight reduction is essential, which is realized using alloys welding process. Application of welded Al construction enabled ones to decrease the airplane weight and increase fuel efficiency [9]. However, the corrosion behavior of aluminum alloys becomes more complicated after the welding process [10,11,12].

Tungsten inert gas (TIG) welding is one of the most popular types of welding, which is used in the aerospace industry and has such advantages as arc stability, good weld forming and pure metal composition [13,14,15]. Therefore, there are studies dealt with the improvement of the TIG process, corrosion properties of the obtained material and its microstructure. Li et al. [16] suggested a special way of direct current electrode negative TIG welding the 2219 Al alloy, which dealt with coating an active agent on the welding joint zone. Lin et al. [15] studied the influence of post-weld heat treatment on the microstructure of the weld metal of variable polarity TIG welded AA2219 joints using crack tip opening displacement test method. Niu et al. [17] used double-pass tungsten inert gas arc welding of 2219-T87 aluminum alloys and studied the distribution influence of alloying elements and precipitations, size of grains and welding temperature field on softening behavior of FZ and HAZ and made a correlation between the mechanical properties and microstructure of the obtained joints. Zhang et al. [14] combined numerical simulation with experimental methods to study the inconsistency in the mechanical properties of 2219 aluminum alloy TIG-welded joints. Chen et al. [13] investigated the microstructure of pure aluminum TIG welds fabricated under ultrasonic fields using continuous and fixed-point welding and established that the applied ultrasound can break and refine the grains of the weld. However, the reasons, initiation, and propagation of severe localized corrosion in TIG joints of Al alloys were not studied in detail.

Queiroz et al. [10] investigated the influence of unequal levels of deformation and fragmentation on the electrochemical response of friction stir welded (FSW) AA2024-T3 by means of electron backscatter diffraction, scanning electron microscopy, global and localized electrochemical techniques. It was established that FSW leads to heterogeneous distribution and fragmentation of coarse intermetallic particles, which initiates the localized corrosion in the thermo-mechanically affected zone. Proton et al. [18] studied the corrosion behavior of FSW joint in 2050-T3 Al-Cu-Li alloy in 1 M NaCl solution and showed the efficiency of post-welding heat treatment on the corrosion activity of the material. In the other work, Proton et al. [19] showed that the nugget of a friction stir welding joint of 2050 Al-Cu-Li alloy was susceptible to intergranular and intragranular corrosion, which was related to the heterogeneity of microstructure revealed on a microscopic scale. These works indicate that to find out the reason of electrochemical activity of the TIG welded joints of Al alloys the corrosion properties of the material should be studied in combination with detailed microstructural analysis.

Aluminum alloys of the Al-Mg-Sc system combine such properties as medium-strength, high corrosion resistance and possess high weldability. In our previous studies [20,21] we have established using localized electrochemical methods that weld interface of 1579 Al alloy is an area, where corrosion starts. At the same time, we have not correlated this specific place of weld interface, where the corrosion process occurred, with the microstructure of the material. To protect samples against severe corrosion destruction and expand the area of its practical application it is necessary to identify the “weakest” place in the structure of the material. Such knowledge will promote the development of the methodology of manufacturing and will help to understand the mechanism of material degradation as well as to find the optimal ways of corrosion protection.

This study is a continuation of the work [20], where preliminary investigations of the electrochemical properties of the welded joint were performed. In the current work to reveal the reason of high electrochemical activity of the weld interface, the microstructure of the material and chemical composition of the specific phases, which are activators of the material corrosion, were studied. The main objective of this work is to establish the role of the intermetallic phases on the Al alloy corrosion. This is very important information, which will help to understand the effect of microstructure, the presence of specific secondary phases in the composition of Al weld on its corrosion activity and as a result to develop the ways of limitation of corrosion propagation.

The corrosion activity of the welded zone was examined using modern informative methods of localized corrosion identification [22,23,24,25,26,27,28,29,30,31,32,33,34]. The microstructure analysis, microhardness distribution along the different zones of 1579 aluminum alloy weld were performed for the first time in correlation with determination of the localized electrochemical activity of the material. Plasma electrolytic oxidation (PEO) [24,27,35,36,37,38,39,40,41,42,43,44,45] of the welded material was applied to inhibit its electrochemical activity.

## 2. Materials and Methods 

### 2.1. Samples

**Figure 1:**
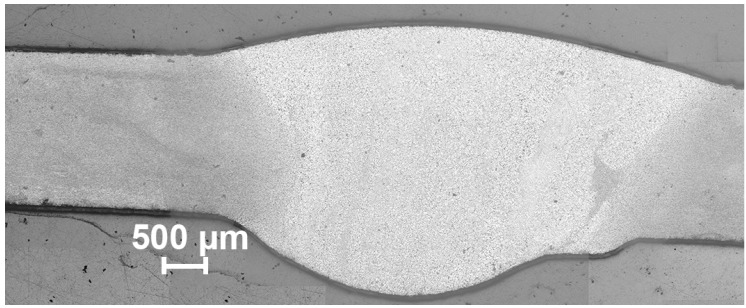
The image of the cross-section of the Al alloy welded joint.

The welded joint of two 1579 Al alloy plates (Figure 1) was a specimen under study. Composition of this aluminum alloy (Al-Mg-Sc system) is presented in Table 1. Tungsten inert gas (TIG) welding process, cross-section formation, and surface mechanical treatment were described in [20]. TIG welding was carried out using high purity argon (shield gas) with the flow rate equaled to 43 ± 3 L min^−1^. The range of interpass temperature was 60–80 °C. Welding current was 175 A.

To reveal boundaries of welded material grains and phases the etching aqueous solution with the following composition was used: HNO_3_ (Sigma-Aldrich, St. Louis, MO, USA) HCl (Sigma-Aldrich, St. Louis, MO, USA), HF (Sigma-Aldrich, St. Louis, MO, USA), and H_2_O, 2.5 mL (65 wt.%), 1.5 mL (35 wt.%), 1 mL (40 wt.%), and 95 mL, respectively. The surface of the welded joint after etching is presented in Figure 1.

The PEO-layer was formed on the welded Al alloy surface at 0.5 A cm^−2^ current density. The electrolyte for PEO consisted of sodium fluoride (NaF) and potassium tartrate hemihydrate (C_4_H_4_O_6_K_2_∙0.5H_2_O, Sigma-Aldrich, St. Louis, MO, USA), 0.3 g L^−1^ and 15 g L^−1^, respectively. The oxidation time was equal to 160 s. The formed coating thickness was about 11 µm.

### 2.2. Electrochemical Measurements

The localized corrosion studies of the 1579 Al alloy weld in correlation with its microstructure were performed for the first time. The electrochemical activity of the bare sample, as well as the anticorrosion properties upgrade after PEO treatment, were studied using Scanning Ion-Selective Electrode Technique (SIET) and Scanning Vibrating Electrode Technique (SVET) on equipment supplied by Applicable Electronics (Applicable Electronics, New Haven, CT, USA). The detailed methodology of SVET-SIET tests was described in [20].

The typical SVET electrode made from Pt-Ir insulated wire coated with a Pt black was used. The distance between the surface of the investigated material and SVET probe was 100 μm. The SVET probe amplitude of vibration was about 17 μm. The SIET pH electrode was used in this work. The SIET microelectrode membrane was defined previously [30]. The pH electrode was backfilled with 0.01 M KH_2_PO_4_ (Sigma-Aldrich, St. Louis, MO, USA) in 0.1 M KCl (Sigma-Aldrich, St. Louis, MO, USA). The distance between the SIET pH electrode and the specimen surface was equal to 40 μm. SVET/SIET tests were performed during 8 hours. Specimens were tested in 3% sodium chloride solution.

### 2.3. Microstructure Characterization

The welded joint zone was studied using inverted microscope Axiovert 40 MAT (Carl Zeiss Group, Oberkochen, Germany). Scanning electron microscopy coupled with energy dispersive X-ray analysis (SEM-EDX) were used to study the sample microstructure and phase composition using Zeiss EVO 40 (Carl Zeiss Group, Oberkochen, Germany). The detailed analysis of microstructure was performed by means of Hitachi S5500 (Hitachi High Technologies Corporation, Tokyo, Japan). SEM images of the surface and cross-section of the PEO-coating formed on the 1579 Al alloy were studied using SIGMA HD (Carl Zeiss Group, Oberkochen, Germany). Accelerating voltage of 10–20 kV was applied to obtain SEM-images. SE2 and BSE detectors were used to obtain SEM images.

To evaluate the grain size of α-matrix and fractions of the secondary phases in various parts of the weld, the images were processed using the ImageJ software (National Institutes of Health, Bethesda, MD, USA).

X-ray diffractometer D8 Advance (Bruker AXS GmbH, Karlsruhe, Germany) was used to confirm the presence of secondary phases in the weld. Since the welding area was narrow, zones outside the weld were cut before the XRD analysis.

### 2.4. Microhardness Test

The microhardness test of 1579 Al alloy weld as well as analysis of the microhardness distribution in HAZ, FZ and BM were performed for the first time. Microhardness of the sample was studied using dynamic ultra-microhardness tester DUH-W201 (Shimadzu, Kyoto, Japan) in load-unload mode. The indenter was Berkovich triangular pyramid with the angle of the tip equal to 115°. Tests were performed at the load of 500 mN and this loading was kept for 5 s. Universal (Martens) microhardness (*HM*) and Indentation Hardness (*H_IT_*) were determined from load-displacement data obtained by indentation.

*HM* and *H_IT_* were calculated using Equations (1) and (2), respectively [46].

*HM* = *F*_MAX_/*A*_S_, (1)

where *F*_MAX_ is the applied maximum load, *A*_S_ is the contact area at *F*_MAX_, which is equal to *A*_S_ = 26.43∙*D*^2^, and *D* is the indenter penetration depth at *F*_MAX_.

*H_IT_* = *F*_MAX_/*A*_P_, (2)

where *F*_MAX_ is the applied maximum load, *A*_P_ is the projected area of the elastic contact at *F*_MAX_, which is equal to *A*_P_ =23.90∙*D*_C_^2^, and *D*_C_ is the contact depth.

## 3. Results and Discussion

**Figure 2:**
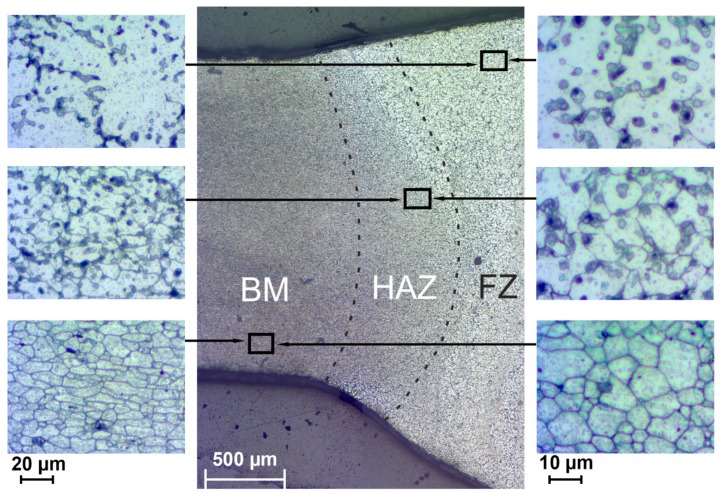
The microstructure of the TIG weld joint of the aluminum alloy including BM, HAZ, and FZ.

After TIG welding of aluminum alloys three zones can be revealed in the material structure (Figure 2): base metal (BM), heat affected zone (HAZ) area, where microstructure evolution is a result of thermal treatment, and fusion zone (FZ), where plastic deformation and recrystallization take place.

The result of the microstructure study of the welded joint is shown in Figure 2. Image analysis enables one to detect three abovementioned zones. BM zone is characterized with small elongated well-defined grains, with the average size (9 ± 3) µm and several precipitates. In the HAZ the average grain size of α-matrix increased up to (16 ± 3) µm. Due to the intensive recrystallization process in FZ, it is very difficult to identify the grain boundary. The grain size of FZ changed in a wide range and its average value was equal to (21 ± 5) µm. Therefore, the grain size directly related to the thermal treatment and recrystallization process and increased in the following order: BM < HAZ < FZ. It should be emphasized that the quantity of precipitation of secondary phases (black spots) differs in all three areas and is maximum in the HAZ. The fraction of secondary phases in three parts of weld was calculated using Figure 2 analysis. The results indicated that BM and FZ have lower fractions of secondary phases (26% and 28%, respectively) as compared to ones in HAZ (38%). This result can predetermine the heterogeneous character of material corrosion distribution.

**Figure 3:**
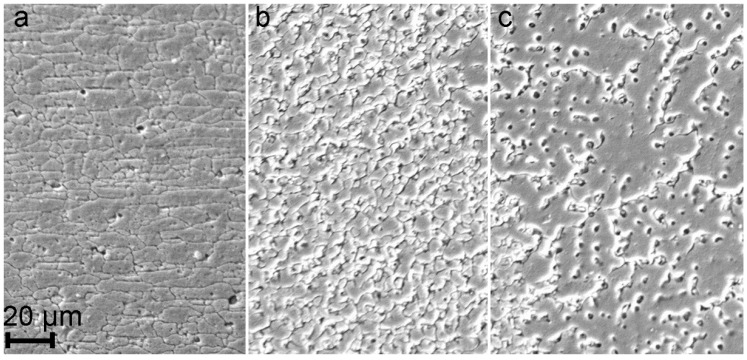
SEM images of BM (**a**), HAZ (**b**), and FZ (**c**) of the 1579 Al alloy weld.

SEM images of obtained structures of 1579 Al alloy weld are presented in Figure 3. The results of the images analysis of BM (Figure 3a), HAZ (Figure 3b), and FZ (Figure 3c) are conformed to data of optical images shown in Figure 2. Due to the highest volume fraction of secondary phases in the heat affected zone and based on previous studies [20,21], an assumption was made about higher corrosion activity of HAZ as compared to BM and FZ.

**Figure 4:**
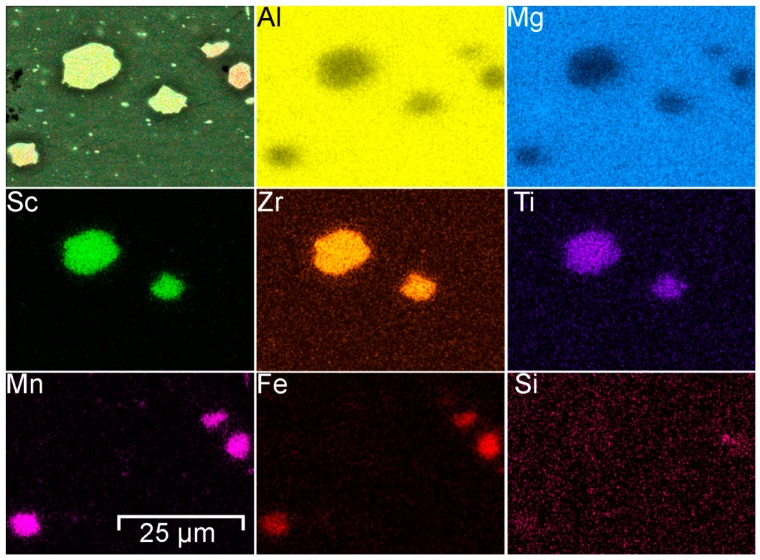
Element distribution in the HAZ. Presence of Al-Sc-Zr-Ti and Mn-Fe-Al phases was detected.

To study the chemical composition of grains and phases SEM-EDX analysis was performed (Figure 4). Analysis of the result indicates that aluminum alloy welded joint contains aluminum matrix grains consist of Al and Mg. various phases and intermetallic compounds were detected in HAZ. These secondary phases were composed of Al-Sc-Zr-Ti and Mn-Fe-Al.

Scandium is the effective material structure modifier and inhibitor of recrystallization for Al alloys [47]. Such behavior of Sc in the Al-Mg alloys provides a significant increase in the strength characteristics while maintaining at a high level the plastic and technological properties of the wrought alloy. This is very important in the manufacture of complex welded structures. This effect is related to the interaction of Sc with Al with the formation of high-strength intermetallic compounds like Al_3_Sc. These particles have a spherical shape and compactly distributed in a matrix. Scandium-containing particles are fully coherent with the Al alloy matrix and are effective blockers of grain boundaries migration. Al_3_Sc-like particles decelerate and in some cases prevent the recrystallization process in wrought alloys and refine the cast grain structures [47,48,49,50]. Decrease of grain size leads to the decrease in corrosion rate values of Al samples [51]. In [52] it was established that Al_3_Sc particles in Al-alloys are comparatively weak local cathodes with low self-dissolution rate and due to small size, their possess good electrochemical compatibility with Al alloys and do not provoke intensive pitting and localized corrosion. Manganese (Mn) and zirconium (Zr), which are usual additives in the industrial aluminum alloys, have similar to Sc the anti-recrystallization property, which ensures the formation of a subgrain structure of Al alloy and contributes to more complete separation of the main strengthening phases [50,53,54]. 1579 alloy belongs to Al-Mg-Sc system and such Sc-containing phases as Al_3_Sc [49], Al_3_(ScZr) [47,55] and Al_3_(ScTi) [56] can be possibly formed in the material (Figure 4).

**Figure 5:**
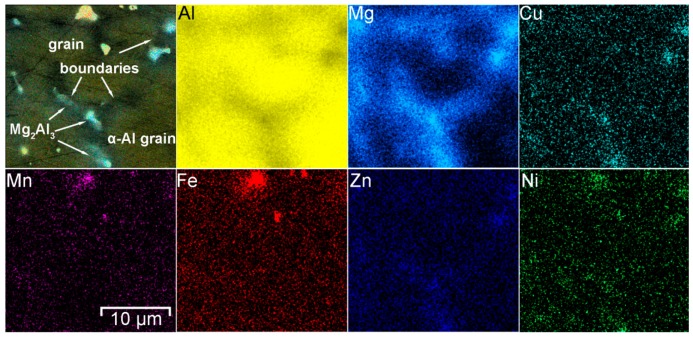
SEM-EDX study of the grain boundary in HAZ. Presence of Mn-Fe-Al and Mg-Al phases was detected.

**Figure 6:**
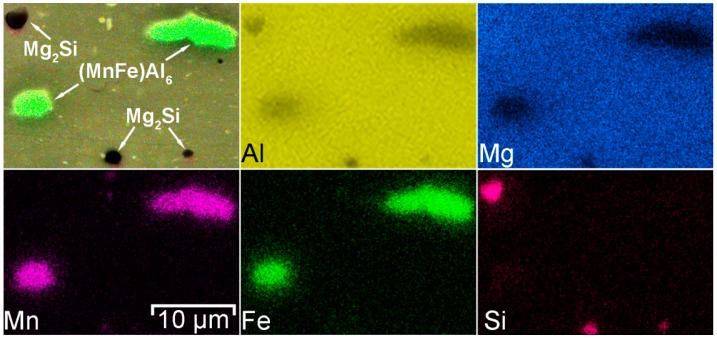
Element distribution in the HAZ. Presence of Mn-Fe-Al and Mg-Si phases was detected.

**Figure 7:**
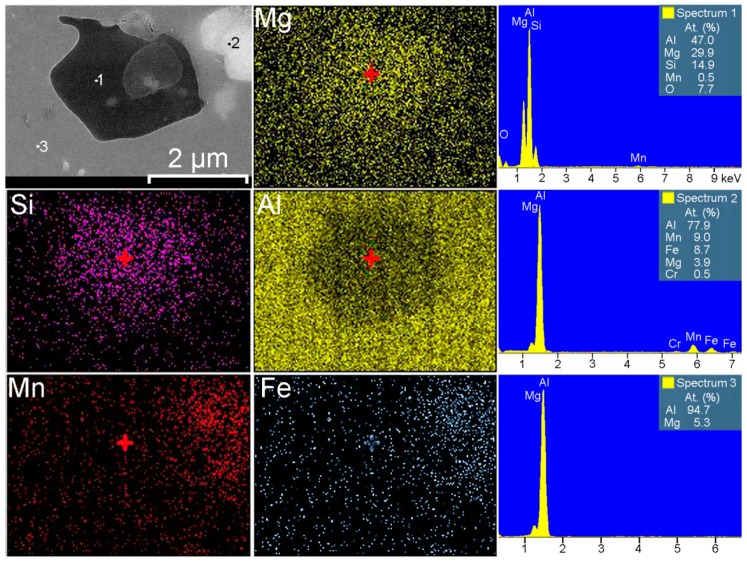
Detailed SEM-EDX analysis of black spots in the HAZ. Presence of Mn-Fe-Al and Mg-Si phases was detected.

Iron (Fe) in the 1579 alloy composition is present as an impurity. Manganese is an alloying element, which binds Fe in such phases as (MnFe)Al_6_ [57,58] or (MnFe)_3_SiAl_12_ [59]. Formation of these phases was confirmed using SEM-EDX analysis (Figure 4, Figure 5, Figure 6 and Figure 7). The EDX data in Figure 5 show that such elements as Ni can substitute for Mn, and the phase Al_6_(NiMnFe) can be formed. Such phase is commonly formed during solidification of the alloy [59].

SEM-EDX analysis of the alloy grain in the HAZ indicated the higher concentration of magnesium near the grain boundary as compared to the bulk aluminum matrix (Figure 5). Due to the high Mg content (6.78 wt.%) in the 1579 alloy, there is a tendency for the Mg_2_Al_3_ intermetallic phases (β-phase) [60] to form along the grain boundaries. This is because the equilibrium solubility of magnesium in aluminum is about 2% at ambient conditions [61]. Spectrum 3 in Figure 7 indicates that α-aluminum alloy grains consist of Al and Mg, 95 at.% and 5 at.%, respectively. Figure 5 also shows the possible presence of (MnFe)Al_6_, Al_2_Mg_2_Zn_3_ (Т-phase) [62,63], AlNi_3_ [64], Al_2_Cu (θ phase) [65], and Al_2_CuMg (S-phase) [65] phases.

1579 alloy also contains silicon as an alloying element, which has a positive effect on welding and brazing process of the material. Formation of Mg_2_Si particles [59,65,66,67] (β-phase) makes silicon-containing Al alloys thermally strengthened. Presence of these phases in high amount was registered by SEM-EDX analysis (Figure 6 and Figure 7). Eutectic Mg_2_Si particles were etched with a solution, which contains hydrofluoric acid and presented in Figure 2 like black spots. Detailed analysis of the black spot showed that it consists of Mg and Si in the atomic ratio 2/1 (Spectrum 1, Figure 7).

According to the results shown in Figure 2 concentration of the black spots increased, when we moved from BM to HAZ and decreased from HAZ to FZ. This is related to the heat impact during the welding process. Mg_2_Si, as well as Mg_2_Al_3_ phases, coalesce, while iron-containing phases remain unchanged, which is mainly affected the corrosion resistance of the HAZ.

In accordance with Table 2 [65], corrosion potential of Mg_2_Si and Mg_2_Al_3_ is lower as compared to pure aluminum. Therefore, these phases act as anode, whereas α-matrix, and especially Fe, Mn, Ti, Zr-containing compounds are cathode. Spectrum 2 in Figure 7 shows that Mn and Fe presented in the phase in the atomic ratio 1/1, which confirmed the possible formation of (MnFe)Al_6_ phase. Such Fe-containing phase like (MnFe)Al_6_ presented in the composition of the welded material is a power cathode, which induces the corrosion activity of the neighboring lower potential phases and aluminum matrix.

Therefore the reason of probable high corrosion activity of the HAZ was attributed to the precipitation of Fe rich secondary phases (e.g. (MnFe)Al_6_) along with Mg_2_Si and Mg_2_Al_3_ phases at the recrystallized grain boundaries (Figure 5, Figure 6 and Figure 7) that stimulate the micro-galvanic corrosion in these zones.

**Figure 8:**
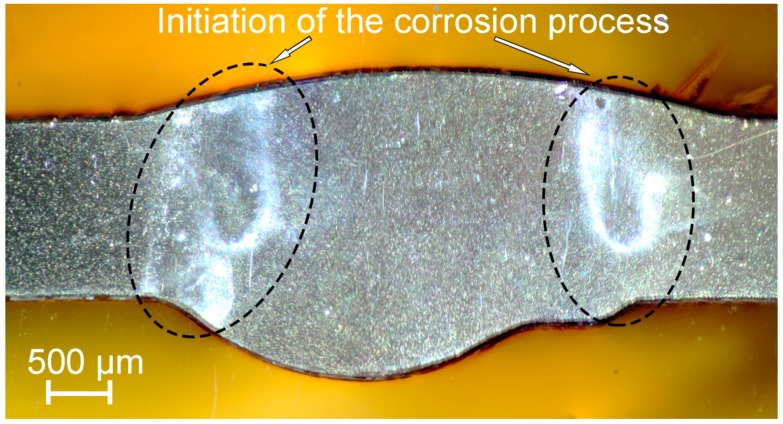
Optical image of the sample after 1.5 h immersion in 3% NaCl. Initiation of the corrosion was detected in the HAZ.

**Figure 9:**
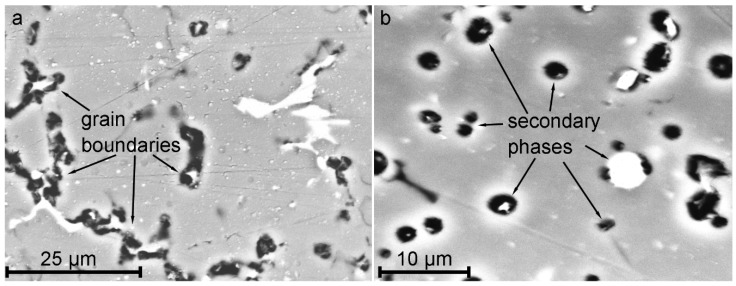
SEM images of HAZ corrosion degradation realized at the grain boundary (**a**) and around cathodic phases (**b**).

**Figure 10:**
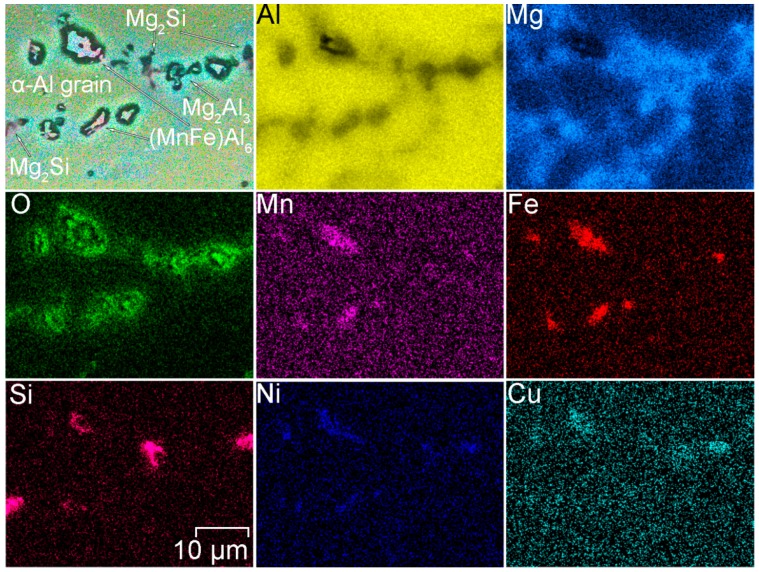
SEM-EDX analysis of the corrosion-active area in the HAZ after 1.5 h immersion in 3% NaCl.

To study the process of corrosion initiation and to exclude the effect of etching-out of anodic zones the sample was immersed in 3% NaCl for 1.5 h. Corrosion starts in HAZ after 30 min of sample exposure and began to propagate along the weld interface (Figure 8). After 1.5 h the sample was removed from the solution rinsed with deionized water and air-dried. SEM-EDX analysis was performed to study the place of heat affected zone, where intensive corrosion propagation was monitored (Figure 9 and Figure 10). SEM images (Figure 9) show the result of corrosion attack of different places of HAZ. Figure 9a depicts the corrosion degradation realized at the grain boundary, whereas Figure 9b indicates the dissolution of the α-aluminum matrix and secondary anodic phases around cathodic ones. Three phases were identified in the place of corrosion activation: (MnFe)Al_6_, Mg_2_Si, and Mg_2_Al_3_. Ni and Cu were also detected in the composition of the material (Figure 10). Therefore, such abovementioned phases as Al_6_(NiMnFe), Al_2_CuMg could be presented in the HAZ. Analysis of the results indicates that corrosion process occurred along the boundary of the α-Al grains, where a high concentration of secondary anodic (Mg_2_Si and Mg_2_Al_3_) and cathodic phases ((MnFe)Al_6_) is presented. Corrosion propagation is confirmed by high oxygen content on the surface of anodic regions. The welding leads to coalescence of the anodic phases in the HAZ that makes this area more susceptible to corrosion. The overall analysis of the size and composition of secondary phases in HAZ performed using SEM-EDX data presented in Table 3. The most frequently distributed phases in heat affected zone are Mg_2_Si, Mg_2_Al_3_, (MnFe)Al_6_, and Al_3_(ScZr). Presence of other elements (like Cr, Ti, Cu, etc.) related to the penetration of X-ray beam through the studied secondary phases to the bulk of the material, which contains these precipitations. XRD analysis confirmed the presence of phases shown in Table 3 in the weld zone of Al alloy.

Our results are similar to [18,19], where HAZ of friction stir welded 2050 alloy underwent serious corrosion attack at grain boundaries in 1 M NaCl solution. Authors in [68] showed that dark spots in the structure of the 2A97 Al alloy welded joint after immersion in a 3.5 wt.% NaCl solution are related to intermetallic particles, which cause the localized corrosion in HAZ. In [69] severe corrosion attack was observed in the heat affected zone of TIG weld joint of AA2014. In [70] it was found the formation of a high amount of corrosion products in the HAZ as compared to other zones of welded joint of 7N01-T5 Al alloy, which indicates the severe corrosion in HAZ. Authors in [71] established galvanic corrosion in welded Al-Zn-Mg alloy joints, where HAZ played the anodic role, while FZ was a cathode. All these results indicate that HAZ is the corrosion active area in the welded joints.

**Figure 11:**
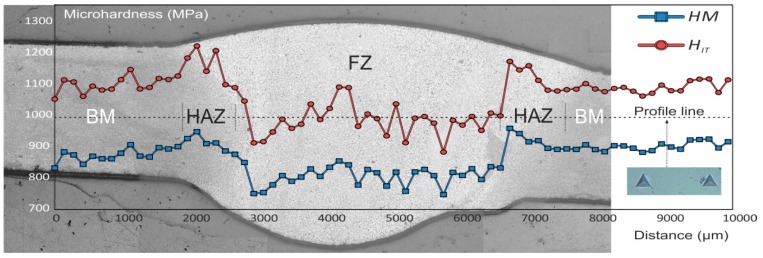
Universal microhardness (*HM*) and Indentation Hardness (*H_IT_*) distribution along BM, HAZ, and FZ of the welded joint.

**Figure 12:**
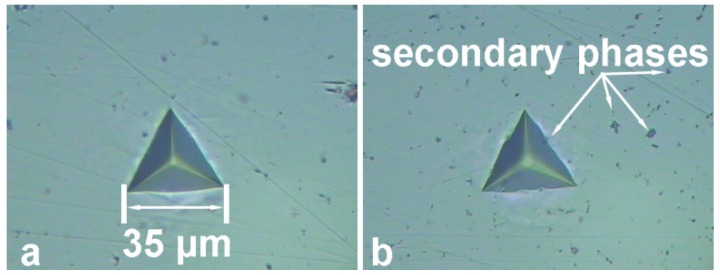
Test indentations in the BM (**a**) and HAZ (**b**).

In order to study the influence of the TIG welding on the mechanical characteristics of the material, the microhardness was measured along the cross-section of the sample. Figure 11 shows the distribution of the Universal microhardness, *HM,* and Indentation Hardness, *H_IT_*, along the BM, HAZ and FZ areas of the joint. Average *HM* and *H_IT_* values of the base metal were equal to 895 MPa and 1100 MPa, respectively. Average *HM* and *H_IT_* values of the HAZ were equal to 930 MPa and 1170 MPA, respectively. Average *HM* and *H_IT_* values of the FZ were equal to 810 MPa and 990 MPa, respectively. It should be noted, that higher *H_IT_* values in comparison with *HM* are related to the elastic deformation of the material at the applied load. The higher microhardness values of the HAZ as compared to those of BM and FZ are related to the enrichment of HAZ with hard Mg_2_Si eutectic phases and (MnFe)Al_6_ intermetallic compounds [72,73], which were confirmed by SEM-EDX analysis. Higher concentration of the hard secondary phases in HAZ as compared to BM is shown in Figure 12b. Presence of intermetallic compounds in the area of test indentation and neighboring zone reveals the reason of higher microhardness values in the HAZ.

**Figure 13:**
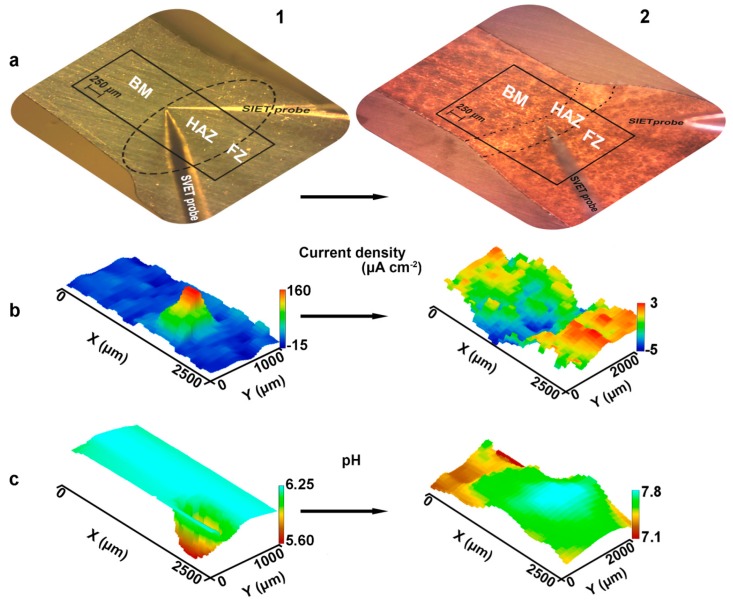
The optical image (**a**), SVET (**b**) and SIET (**c**) 3D maps of the studied welded joint area including BM, HAZ, and FZ for the sample without (**1**) and with PEO-coating (**2**). SVET and SIET maps presented after 8 h of sample exposure to 3% NaCl solution.

To correlate the microstructure of the welded joint with its electrochemical activity additional precision SVET-SIET tests were carried out (Figure 13 1a–1c). To determine the exact place of corrosion propagation in the weld interface the SVET-SIET scans include all three zones: BM, HAZ, and FZ. During the experiment (8 h) high values of current density (up to 160 µA cm^−2^) were established in the HAZ, where low pH was also detected (down to 5.6). In accordance with the work [20] these data indicate the high susceptibility of HAZ to corrosion degradation, the corrosion process development begins in this area. This result confirms the immersion tests presented in Figure 8 and the assumption about severer corrosion of HAZ as compared to BM and FZ. The corrosion mechanism is related to abovementioned secondary phases presence and their high concentration in the HAZ. These phases probably have more positive potential as compared to α-matrix. Therefore, such phases are cathodes, which accelerate the dissolution of the material in the neighboring area. According to [74] the area of boundaries between phases and grains has the lowest Volta potential and suffers from severe micro-galvanic corrosion.

**Figure 14:**
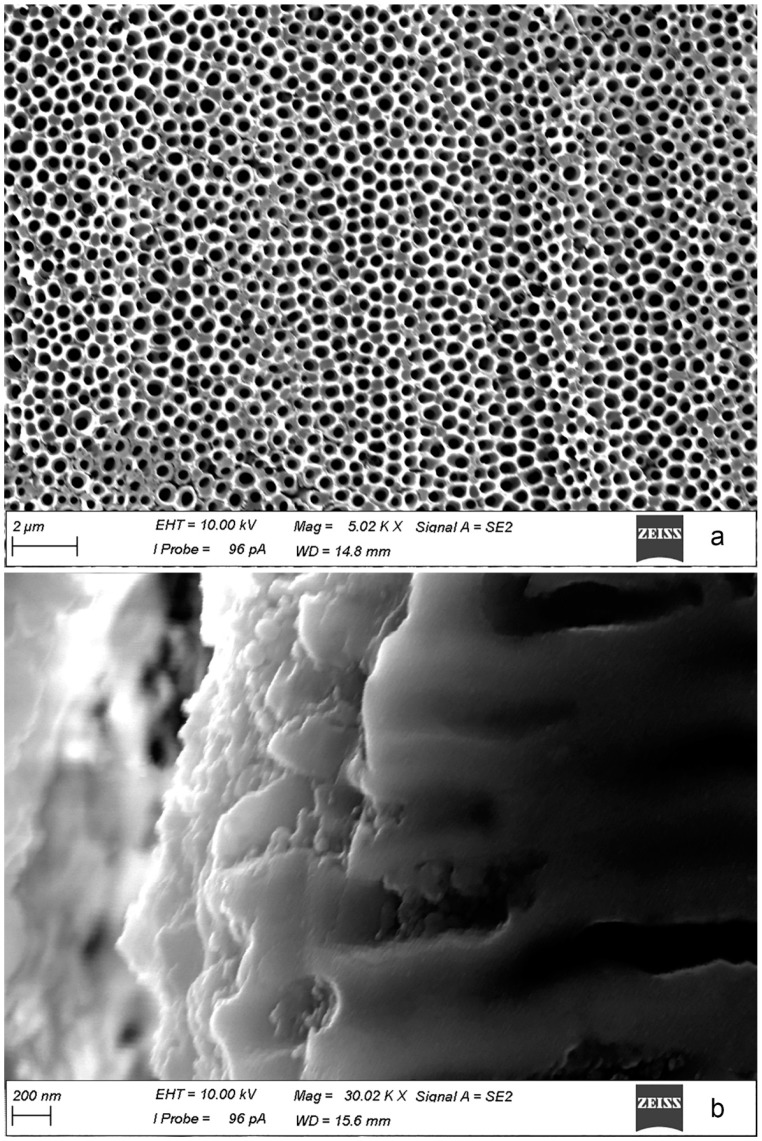
SEM images of the surface (**a**) and cross-section (**b**) of the PEO-coating formed on 1579 Al alloy weld.

PEO method was used to decrease the activity of the weld interface and make the joint more corrosion resistant. SEM images of the surface and cross-section of obtained PEO-coating on the surface of 1579 Al alloy weld are presented in Figure 14. Formed PEO-layer has a convoluted structure composed of microtubes with an average pore size of 390 ± 80 nm (Figure 14a). Cross-section presented in Figure 14b shows the structure of the topmost part of the PEO-coating, which contains microtubes. SVET and SIET were used to identify the intensity of the electrochemical process on the surface of the welded joint after PEO treatment (Figure 13 2a–2c). During the exposure to NaCl solution (8 h) the PEO-coated sample was stable and activation of the corrosion process was not detected. PEO treatment of the sample sufficiently reduces the current density measured on the material surface and slightly shifts pH to the more alkaline range (Figure 13 2b,2c). It should be noted, that according to SVET/SIET data HAZ became a cathodic zone (blue area) after PEO treatment. This effect is probably related to features of the microstructure of HAZ, with the presence of a high amount of the aforementioned secondary phases, which promote the formation of denser PEO-coating as compared with other parts of the sample, where low anodic activity (red area) could be found. This result confirmed our previous corrosion tests [21] and indicates the improvement of the corrosion properties of the welded joint and especially HAZ after protective coating formation. 

## 4. Conclusions

In summary, the microstructure of the TIG Al-Mg-Sc alloy welded joint has been studied in correlation with localized corrosion activity. Analysis of the result leads to the following conclusions:

1. It has been established that HAZ is more sensitive to localized corrosion as compared to the fusion zone and base material. The higher electrochemical activity of HAZ is caused by precipitation of cathodic Fe rich secondary phases (e.g. (MnFe)Al_6_) along with anodic Mg_2_Si and Mg_2_Al_3_ phases at the recrystallized grain boundaries that stimulate the micro-galvanic corrosion in these areas due to the different corrosion potential;
2. During heat treatment realized in TIG welding, Mg_2_Si particles, as well as Mg_2_Al_3_, coalesce, while iron-containing phases remain unchanged, which is mainly affected the corrosion resistance of HAZ. The microstructure analysis of the welded joint showed that concentration of the black spots, which are Mg_2_Al_3_, Mg_2_Si phases increased in HAZ and decreased in BM and FZ. SEM-EDX analysis showed the intensive intergranular corrosion in HAZ, where a high concentration of (MnFe)Al_6_, Mg_2_Si, Mg_2_Al_3_ particles was detected;
3. It has been established the higher microhardness values of HAZ as compared to those of BM and FZ, which is related to the enrichment with hard Mg_2_Si phase and hard (MnFe)Al_6_ intermetallic compounds;
4. According to SVET and SIET, the coating, obtained using PEO treatment reduces the electrochemical activity of the welded joint and inhibits its corrosion degradation. This method enables one to improve the corrosion properties of the welded material.

## Figures and Tables

**Table 1:** The concentration of the alloying elements in the composition of the alloy.

Element	Al	Mg	Si	Mn	Zn	Cr	Fe	Cu	Sc	Zr	Ni	Ti
Wt.%	balance	6.78	0.51	0.30	0.62	0.17	0.15	0.14	0.13	0.13	0.10	0.02

**Table 2:** Corrosion potential of pure aluminum and Al alloy secondary phases in 0.6 M NaCl solution [65].

Phases	Al	Mg_2_Al_3_	Mg_2_Si	Al_3_Fe	Al_6_Mn	Al_3_Ti	Al_3_Zr
Corrosion potential, mV_SCE_	−849	−1162	−1536	−566	−913	−799	−801

**Table 3:** Size and composition of α-Al matrix grain and secondary phases in HAZ of 1579 Al alloy weld.

	Phases	α-Al	Mg_2_Al_3_	Mg_2_Si	(MnFe)Al_6_	Al_3_(ScZr)
	Size, µm	16 ± 3	3 ± 1	2 ± 1	6 ± 3	9 ± 2
EDX analysis, at.%	Al	94.7	59.2	47.0	77.9	58.1
	Mg	5.3	38.9	29.9	3.9	–
	Si	–	–	14.9	–	–
	Mn	–	–	0.5	9.0	–
	Fe	–	–	–	8.7	–
	Sc	–	–	–	–	18.9
	Zr	–	–	–	–	18.7
	Ti	–	–	–	–	4.3
	Cr	–	–	–	0.5	–
	Zn	–	1.4	–	–	–
	Cu	–	0.5	–	–	–
	O	–	–	7.7	–	–
